# Significant Deficits Are Seen in Extensor Muscle Performance but Not Flexor Muscle Performance 6 Months After Anterior Cruciate Ligament Reconstruction Using a Rectus Femoris Tendon Autograft

**DOI:** 10.1002/ars2.70024

**Published:** 2026-05-11

**Authors:** Alef Cavalcanti Matias de Barros, Márcio Cabral Fagundes Rêgo, Karinna Sonálya Aires da Costa Oliveira, Marcelo Cabral Fagundes Rêgo, Carlos Eduardo da Silveira Franciozi, Diego Ariel de Lima, Camilo Partezani Helito, José Leonardo Rocha de Faria, Jamilson Simões Brasileiro

**Affiliations:** ^1^ Department of Physiotherapy Federal University of Rio Grande do Norte (UFRN) Natal Brazil; ^2^ Hospital Memorial São Francisco Natal Brazil; ^3^ Departamento Médico do América Futebol Clube Natal Brazil; ^4^ Departamento de Ortopedia Universidade Federal de São Paulo (UNIFESP) São Paulo Brasil; ^5^ Universidade Federal Rural do Semi‐Árido (UFERSA) Mossoró Brasil; ^6^ Grupo de Joelho Instituto de Ortopedia e Traumatologia, Faculdade de Medicina da Universidade de São Paulo (USP) São Paulo Brasil

## Abstract

**Purpose:**

To evaluate bilateral isokinetic performance symmetry of knee extensors and flexors 6 months after anterior cruciate ligament reconstruction using a rectus femoris tendon autograft.

**Methods:**

This retrospective case series included patients consecutively recruited at a single tertiary sports medicine center between January 2023 and July 2024. Inclusion required primary anterior cruciate ligament reconstruction with a rectus femoris autograft, no prior knee surgeries, and a minimum 6‐month follow‐up; patients with revision or multiligamentous procedures were excluded. Isokinetic testing was performed at 6 months using a computerized dynamometer. The nonoperative limb was tested first, followed by the operative limb. Patients performed 5 maximal effort trials at 60°/s for both extension and flexion. Measured outcomes included peak torque (PT), PT normalized to body weight, angle at PT, total work (TT), and mean power (PM) for both knee extension and flexion. Paired t‐tests compared operated and nonoperated limbs. Statistical significance was set at *P* ≤ .05.

**Results:**

Thirty‐one male patients (mean age, 26.3 ± 4.7 years) were included, with a mean follow‐up of 6.2 ± 0.4 months. At 6 months, the operated limb achieved 71% of contralateral PT and 75% of contralateral PM, with significant deficits in extension PT (*P* < .001), PT normalized to body weight (*P* < .001), TT (*P*  < .001), and PM (*P* = .002), while no significant difference was found for angle at PT (*P* = .457). Flexor performance showed near‐complete recovery, with the operated limb reaching 93% of contralateral peak torque and 90% of TT, with *P* = .355, .340, .902, .153, and .316 for PT, PT normalized to body weight, angle at PT, TT, and PM, respectively.

**Conclusions:**

At 6 months, anterior cruciate ligament reconstruction with a rectus femoris autograft was associated with complete recovery of knee flexor performance but persistent deficits in knee extensor performance.

**Level of Evidence:**

Level IV, retrospective case series.

## Graphical abstract

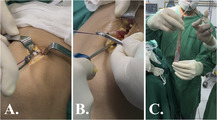

Studies have revealed that an early return to sport (RTS) following anterior cruciate ligament (ACL) reconstruction is associated with a significantly increased risk of graft rupture.^1^ Athletes who resume high‐demand pivoting activities before 9 months postoperatively are up to 7 times more likely to experience reinjury than those who delay their return beyond this threshold.^2^ Furthermore, Grindem et al.^3^ reported that each month of delayed RTS up to 9 months reduces the risk of reinjury by approximately 51%. These findings have led to a growing consensus that clearance for RTS should not be based solely on time, but rather on functional recovery benchmarks, including performance symmetry and neuromuscular control. In our setting, return to pivoting or contact sports is not authorized before 9 months and requires completion of functional testing and clinical clearance.

Despite these recommendations, many patients still undergo routine muscle performance evaluations at 6 months postoperatively—often a key time point for clinical decision‐making. Existing studies at this stage have primarily focused on bone–patellar tendon–bone (BPTB) and hamstring tendon (HT) autografts and indicate that, at ∼6 months, BPTB cohorts commonly exhibit persistent quadriceps (extensor) strength deficits, whereas HT cohorts more often show hamstring (flexor) deficits (with many patients not yet achieving ≥90% quadriceps limb symmetry index).^4^, ^5^, ^6^, ^7^ Limited data are available regarding muscle performance recovery with alternative grafts, such as the superficial layer of the quadriceps tendon (QT)—specifically the rectus femoris (RF) tendon—which has recently emerged as a viable autograft option due to its sufficient length, favorable biomechanical profile, and preservation of the deeper extensor mechanism.^8^

The purpose of this study was to evaluate bilateral isokinetic performance symmetry of knee extensors and flexors at 6 months after ACL reconstruction using an RF tendon autograft. We hypothesized that significant asymmetries in knee extensor performance would persist at 6 months postoperatively, whereas knee flexor performance would show no relevant side‐to‐side differences.

## METHODS

### Study Design and Ethical Approval

Ethical approval was received from the institutional review board, and patients provided informed consent before being enrolled after surgery. Consecutive patients who had undergone primary ACL reconstruction using an ipsilateral RF tendon autograft at a single tertiary sports medicine referral center were retrospectively identified.

Inclusion criteria were patients aged ≥18 years, no prior knee surgeries, no contralateral ACL reconstruction, and no degenerative joint disease or multiligamentous injuries (including lateral extra‐articular tenodesis or other ligament reconstructions). Exclusion criteria were revision ACL surgery or graft harvesting from the contralateral limb and any concomitant procedure requiring meniscal repair or chondral surgery.

Recruitment occurred during routine outpatient follow‐up between 1 and 5 months postoperatively, and testing was scheduled at ∼6 months.

### Surgical Technique

All procedures were performed by 2 fellowship‐trained knee surgeons (M.C.F.R., M.C.F.R.) using a consistent arthroscopic technique.

#### Graft Harvest

**FIGURE 1:**
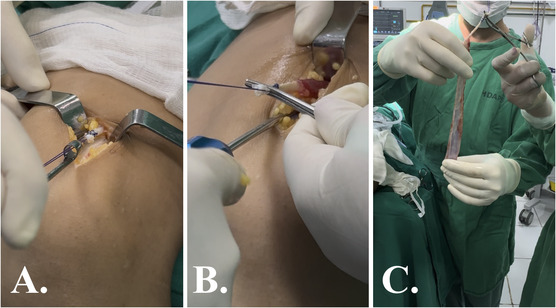
Graft harvest: rectus femoris. (A) Skin incision over the superior pole of the patella at the junction between the lateral and middle thirds for rectus femoris tendon harvest. A cleavage plane is developed approximately 3 cm proximal to the patella. A 10‐mm‐wide graft is outlined with 2 parallel incisions in the superficial layer and detached distally from the patella. The free end is whipstitched with nonabsorbable sutures. (B) The dissection is extended proximally for approximately 8 cm using scissors, preserving the intermediate and deep layers. With the knee flexed at 20°, the graft is harvested using a closed tendon stripper. Proximally, the rectus femoris lamina broadens; harvest proceeded within this laminar plane using a closed tendon stripper under direct tactile guidance, thereby preserving the intermediate and deep quadriceps tendon layers. No specialized instrumentation was required—once the correct cleavage plane between the rectus femoris and the remaining quadriceps was identified, a standard closed stripper permitted complete proximal release and retrieval of the graft at the desired width. (C) Rectus femoris.

**FIGURE 2:**
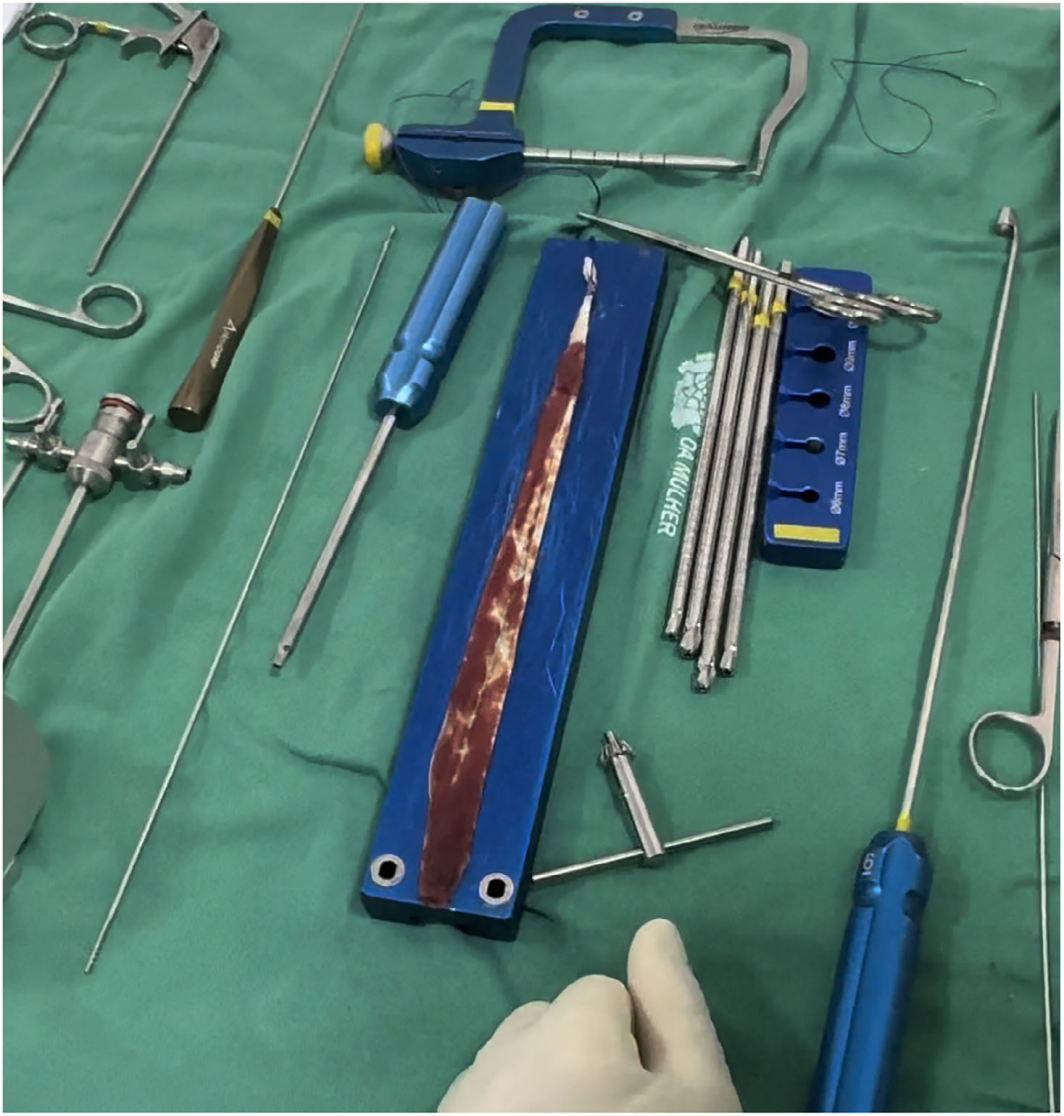
Graft harvest: rectus femoris. The harvested rectus femoris tendon is placed on the preparation table. Excess muscle fibers and irregularities are removed.

The RF tendon graft was harvested using a minimally invasive approach as previously described in the literature^8^ (Figures 1 and 2). A longitudinal skin incision, approximately 3 cm in length, was made beginning at the superior pole of the patella along the lateral third of the QT. The superficial lamina of the RF was carefully identified and dissected from the underlying layers over a proximal length of roughly 8 cm. Care was taken to preserve the intermediate and deep fibers of the quadriceps complex. The knee was maintained in 20° of flexion during harvest, and a closed tendon stripper was used to detach the proximal portion of the graft. The distal free end was whipstitched using nonabsorbable sutures. The harvested graft measured at least 9 cm in length and 8 mm in diameter and was folded asymmetrically. Closure of the harvest site was performed in anatomical layers.

#### ACL Reconstruction

Arthroscopic reconstruction was initiated through a high anterolateral portal and a low anteromedial portal. The femoral tunnel was created using an outside‐in technique, targeting the anatomical footprint of the ACL on the lateral femoral condyle, just posterior to the resident's ridge.^9^ A 90° femoral aiming guide was used to position the tunnel, with external alignment guided by a small lateral incision over the epicondylar area, approximately 4 mm posterior and 8 mm proximal to the lateral epicondyle—corresponding to the anatomical origin of the anterolateral ligament.^10^, ^11^

For tibial tunnel creation, a guide was set at 55° and introduced through the anteromedial portal, with the intra‐articular tip placed at the native tibial ACL insertion, anterior to the posterior cruciate ligament and adjacent to the anterior horn of the lateral meniscus.^9^ Extra‐articularly, the guide was aligned roughly 4 cm below the joint line, 1 cm above the pes anserinus, and 2 cm medial to the tibial tuberosity. A 2‐mm guidewire was advanced under arthroscopic visualization to establish the tunnel path.

The graft was passed from the tibial to the femoral tunnel using a suture shuttle. Femoral fixation was achieved with an interference screw matching the tunnel diameter, while tibial fixation was secured with an interference screw one size larger, applied with the knee in 20° of flexion to ensure proper tensioning.

### Postoperative Rehabilitation

All patients followed a standardized, time‐based rehabilitation program supervised by the same institutional physical therapy team; the program was identical for both graft groups. Early rehabilitation (postoperative days 0‐14) emphasized range‐of‐motion restoration and quadriceps activation, with weight‐bearing as tolerated and typical discontinuation of crutches by week 2.

From weeks 4 to 6, progressive strengthening (closed‐kinetic‐chain) and neuromuscular training were advanced. Between weeks 6 and 12, lower‐extremity strengthening and proprioceptive training were intensified, with low‐impact cardiovascular conditioning maintained throughout. Straight‐line running was typically introduced between months 4 and 5, followed by light plyometrics and change‐of‐direction drills around months 5 to 6.

Return to pivoting sports was allowed no earlier than 9 months postoperatively, contingent on completion of sport‐specific functional testing and clinical clearance.

The rehabilitation protocol was identical for both graft groups to minimize variability in recovery outcomes. Isokinetic testing was not used to guide clinical rehabilitation or RTS clearance; it was performed retrospectively at 6 months specifically for research purposes.

### Outcome Measures and Testing Protocol

Isokinetic muscle performance testing was performed in a single morning session for all participants, using a standardized protocol. Muscle performance assessments were performed on both the operated and contralateral limbs to determine postoperative performance differences. They were conducted at a single postoperative time point, approximately 6 months following surgery. To control for fatigue and order effects, the nonoperated limb was consistently assessed first, followed by the operated limb. Participants were instructed to avoid alcohol consumption, ergogenic aids, and strenuous physical activity for at least 48 h prior to testing.

Muscle performance of both the knee extensors and flexors was assessed using a computerized isokinetic dynamometer (Multi‐Joint System 4, Biodex, Shirley, NY, USA). The following variables were recorded for each muscle group: peak torque (PT), PT normalized to body weight, angle at PT, total work (TT), and mean power (PM).

Before testing, subjects performed a 10‐min warm‐up on a stationary bicycle at a self‐selected pace. They were then positioned on the dynamometer seat, with stabilization straps applied to the trunk and pelvis. The knee joint axis was aligned with the dynamometer axis using the lateral femoral condyle as a reference. The resistance arm was attached and secured 5 cm proximal to the medial malleolus in accordance with manufacturer guidelines.

Participants first performed 3 submaximal concentric contractions of knee extension and flexion at 60°/s for familiarization. This was followed by 5 maximal concentric repetitions at the same velocity, with movements performed from 90° of knee flexion to full extension. After a rest interval of 2 min, the same protocol was applied to the contralateral limb. Throughout testing, standardized verbal encouragement and real‐time visual feedback were provided to ensure maximal effort. For each outcome measure, the maximum value recorded across the 5 maximal trials was used in the statistical analysis.

The test protocol allowed for comparative evaluation of both extension and flexion muscle performance in the operated and nonoperated limbs, thereby enabling analysis of postoperative muscle symmetry in both functional planes.

### Statistical Analysis

Data analysis was conducted using SPSS software version 20.0 (IBM, Armonk, NY, USA). Descriptive statistics were calculated and presented as means with standard deviations and 95% confidence intervals. The distribution of variables was evaluated using the Shapiro‐Wilk test to verify normality, while the Levene test was applied to assess the homogeneity of variances between limbs.

Sample size estimation was based on the primary outcome—peak isokinetic quadriceps torque—assuming a standard deviation of 10%, in line with previously published data on post‐ACL reconstruction athletes.^12^, ^13^

A minimal clinically important difference of 10% (Cohen's *d* = 1.0) was used in the power analysis. A total of 30 participants was determined to provide more than 99% power to detect meaningful side‐to‐side differences at a significance level of *α* = 0.05.

Paired sample t‐tests were used to compare isokinetic muscle performance outcomes—both extensor and flexor parameters—between the operated and nonoperated limbs. This approach allowed for direct within‐subject analysis of muscle performance symmetry following ACL reconstruction with a RF autograft. The level of statistical significance was defined as *P* ≤ .05 for all comparisons.

## RESULTS

A total of 31 male patients who underwent ACL reconstruction using an ipsilateral RF tendon autograft were included in the study. The tests were performed at 6 months postoperatively (6.2 ± 0.4 months, range 6.0‐7.0 months). The mean age of participants was 26.3 ± 4.7 years. No female patients met the inclusion criteria during the study period.

### Knee Extensor Muscle Performance: Operated Versus Nonoperated Limb

At 6 months postoperatively, the operated limb showed significant deficits in quadriceps performance compared with the contralateral side. PT, normalized torque, TT, and PM were all significantly reduced (all *P* < .01), whereas the angle at PT did not differ significantly (*P* = .457) (Table 1). On average, the operated limb achieved 71% of contralateral PT and 75% of contralateral PM, underscoring persistent extensor deficits.

**TABLE 1:** Comparison of Operated and Nonoperated Limbs in Knee Extensor Performance

Variable	Operated Limb (Mean ± SD)	Nonoperated Limb (Mean ± SD)	95% CI	*P* Value
PT (Nm)	173.0 ± 62.0	242.4 ± 46.1	−96.6 to −42.2	<.001
PT/BW (%)	217.5 ± 81.4	305.4 ± 68.0	−125.2 to −50.6	<.001
aPT (°)	62.2 ± 9.7	63.7 ± 5.5	−5.4 to 2.4	.457
TT (J)	820.8 ± 294.0	1096.8 ± 227.2	−406.8 to −145.2	<.001
PM (W)	118.1 ± 44.0	153.3 ± 36.7	−55.4 to −15.1	.002

*Note*: Values are presented as mean ± standard deviation. Statistically significant reductions were observed in the operated limb for PT, PT/BW, TT, and PM (*P * < .05). Paired t‐test was used for all comparisons.

aPT, angle at peak torque (°); CI, confidence interval; PM, mean power (W); PT/BW, peak torque normalized to body weight (%); PT, peak torque (Nm); TT, total work (J).

### Knee Flexor Muscle Performance: Operated Versus Nonoperated Limb

In contrast, no significant side‐to‐side differences were observed for knee flexor performance at 6 months (all *P* > .15; Table 2). The operated limb achieved 93% of contralateral PT and 90% of contralateral TT, indicating near‐complete recovery of flexor symmetry.

**TABLE 2:** Comparison of Operated and Nonoperated Limbs in Knee Flexor Performance

Variable	Operated Limb (Mean ± SD)	Nonoperated Limb (Mean ± SD)	95% CI	*P* Value
PT (Nm)	107.9 ± 32.5	115.4 ± 30.3	−23.4 to 8.5	.355
PT/BW (%)	135.3 ± 40.1	144.9 ± 38.8	−29.7 to 10.4	.340
aPT (°)	39.0 ± 14.2	38.6 ± 9.95	−5.8 to 6.6	.902
TT (J)	564.8 ± 176.5	627.6 ± 165.2	−149.7 to 24.0	.153
PM (W)	76.8 ± 25.3	83.4 ± 25.6	−19.6 to 6.4	.316

*Note*: Values are presented as mean ± standard deviation. No statistically significant differences were observed between the operated and nonoperated limbs for any flexor muscle performance parameter (*P* > .05). Paired t‐test was used for all comparisons.

aPT, angle at peak torque (°); CI, confidence interval; PM, mean power (W); PT/BW, peak torque normalized to body weight (%); PT, peak torque (Nm); TT, total work (J).

## DISCUSSION

The primary findings of this study reveal that, 6 months after ACL reconstruction using a RF autograft, patients continued to exhibit significant deficits in knee extensor muscle performance in the operated limb when compared with the contralateral side. In contrast, flexor performance was largely symmetrical between limbs, with no statistically significant differences observed across the measured variables.

Significant reductions were found in PT, TT, and PM in the operated limb compared with the nonoperated side. These deficits are clinically relevant, as insufficient quadriceps performance has been associated with altered landing mechanics, diminished functional performance, and elevated risk of graft failure during RTS phases.^14^, ^15^, ^16^, ^17^ Although the RF graft preserves part of the quadriceps mechanism, our findings indicate that neuromuscular recovery of the extensor apparatus remains incomplete at this stage, echoing similar patterns observed with patellar and HT grafts.^12^, ^14^, ^18^ This underscores the importance of evaluating muscle performance objectively, rather than relying solely on chronological time, when making return‐to‐activity decisions.^15^, ^16^, ^19^, ^20^

In contrast to the persistent extensor deficits, knee flexor performance appeared to normalize by 6 months postoperatively. No significant differences were detected between the operated and nonoperated limbs in any measured flexor parameter, including PT, PT normalized to body weight, TT, PM, or angle at PT. This likely reflects the intact hamstring integrity following RF graft harvest. These findings align with the existing literature, including Slone et al.,^21^ who concluded that QT autografts are “a safe, reproducible, and versatile graft with clinical outcomes equivalent to bone–patellar tendon–bone (BPTB) or hamstring tendon (HT) grafts, while reducing donor‐site morbidity.” Thus, the early restoration of flexor muscle performance supports the use of grafts that spare hamstring function without compromising safety or functional recovery.^22^, ^23^

Coombs and Cochrane^24^ reported an average 23% deficit in peak knee flexor strength—both eccentrically and concentrically—even 12 months after ACL reconstruction using semitendinosus and gracilis graft. Their conclusion reinforces the notion that harvesting HTs can delay recovery of flexor performance, which may be especially detrimental in sports that rely heavily on posterior chain function. These include sprinting, soccer, rugby, basketball, and jumping events in track and field, where the hamstrings play a critical role in high‐speed running, deceleration, and explosive movements. Therefore, selecting grafts that preserve hamstring integrity—such as the RF—may offer strategic benefits for athletes whose performance depends on hamstring power, endurance, and symmetry.

Although the RF tendon has been proposed as a promising alternative for ACL reconstruction due to its anatomical length, ease of harvest, and theoretical preservation of quadriceps function, our findings suggest that, at 6 months, recovery with RF autograft resembles patterns previously reported for other graft types, but no conclusions regarding faster or slower recovery can be drawn without longitudinal or comparative data. This aligns with prior studies reporting persistent quadriceps deficits at 6 to 12 months regardless of graft type, including quadriceps, hamstring, and BPTB autografts.^18^ At 6 months, these similarities indicate that, despite surgical and anatomical advantages, the RF autograft follows a comparable neuromuscular recovery timeline and should not be considered a means to expedite RTS.^12^, ^25^, ^26^ Clinical outcomes have also been reported to be comparable in terms of knee stability, functional recovery, and graft failure rates when QT—including its RF component—is compared with HT and BPTB grafts.^27^ Instead, our data reinforce the notion that muscle function, not graft type alone, should dictate rehabilitation progressions and clearance decisions.

Quadriceps muscle performance has consistently been identified as a critical determinant of safe RTS following ACL reconstruction. Persistent deficits in extensor muscle performance, as observed in our cohort, have been associated with compensatory movement patterns, reduced functional performance, and a significantly increased risk of reinjury. Multiple studies recommend a limb symmetry index of at least 90% in quadriceps PT before considering return to pivoting or contact sports.^14^, ^17^ Our findings reinforce this threshold, as the mean values in the operated limb fell well below the contralateral side across multiple isokinetic parameters. In fact, with the operated limb achieving on average only 71% of contralateral PT, our cohort fell well below the 90% limb symmetry index threshold frequently recommended for safe return to pivoting or contact sports. This highlights the inadequacy of time‐based criteria alone, such as the commonly used 6‐month mark, and underscores the importance of objective, strength‐based assessments in guiding rehabilitation milestones and RTS decisions.^12^, ^17^ Without sufficient quadriceps recovery, athletes may return to play prematurely, increasing the likelihood of graft failure and long‐term functional impairment.

Our findings are consistent with reports that quadriceps deficits often persist at 6 months regardless of graft choice.^7^, ^14^ Although our rehabilitation program was structured and emphasized progressive overload, including phased resistance training and neuromuscular control, the persistence of extensor asymmetry highlights the possibility that greater intensity or longer duration of strength‐focused interventions may be required. This observation underscores the importance of individualized rehabilitation strategies that go beyond standardized time‐based protocols.

This study focuses exclusively on novel data from patients who underwent reconstruction using an RF autograft, a graft type that remains underrepresented in literature on muscle performance recovery. By simultaneously evaluating both extensor and flexor symmetry, we provide a more nuanced understanding of early postoperative recovery patterns. Our results support the growing consensus that RTS decisions should not be guided by time alone, but rather by individualized functional assessments. Future research should investigate the longitudinal recovery curve beyond 6 months, incorporate functional testing (e.g., hop tests and EMG), and include a broader athletic population to validate these findings across sport types and performance demands.

### Limitations

This study has several limitations. First, its retrospective nature creates a risk for selection bias. Second, the cohort consisted exclusively of male patients, which restricts generalizability to female patients; this reflects the referral pattern at our center during the study period. Third, we evaluated only a single postoperative time point at approximately 6 months, without longitudinal follow‐up or preoperative baseline data. Fourth, we did not collect patient‐reported outcome measures or RTS rates at this time point. Fifth, the sample size was modest, which limits precision and precluded subgroup analyses (e.g., sport type or injury patterns). Finally, the absence of a comparator graft group and additional functional tests (e.g., hop tests and proprioception) limits broader extrapolation of the findings.

## CONCLUSIONS

At 6 months, ACL reconstruction with an RF autograft was associated with complete recovery of knee flexor performance but persistent deficits in knee extensor performance.

## DISCLOSURES

The authors (A.C.M.B., M.C.F.R., K.S.A.C.O., M.C.F.R., C.E.S.F., D.A.L., C.P.H., J.L.R.F., J.S.B.) declare that they have no known competing financial interests or personal relationships that could have appeared to influence the work reported in this article.
